# Bioabsorbable Polydioxanone Mesh for Soft Tissue Reinforcement in Revisional Breast Surgery

**DOI:** 10.1093/asjof/ojac004

**Published:** 2022-02-02

**Authors:** Sergey Y Turin, Karol Gutowski

## Abstract

**Background:**

The need for revision procedures after breast implant surgery often arises from the failure of soft tissues to provide a stable implant pocket. Meshes or dermal matrices have been used to reinforce the implant’s soft tissue support. Durasorb (Surgical Innovation Associates, Chicago, IL) is a resorbable polydioxanone (PDO) mesh indicated for soft tissue reinforcement. Its monofilament, macroporous design allows for ease of handling and rapid tissue incorporation. The extended timeline of PDO bioabsorption provides support during the critical portions of soft tissue healing while avoiding long-term complications of permanent products.

**Objectives:**

Evaluate the efficacy of Durasorb PDO mesh for soft tissue reinforcement in revision breast surgery.

**Methods:**

This is a prospective case series of 17 patients (27 breasts) undergoing revision breast implant surgery with Durasorb PDO mesh.

**Results:**

Five patients (5 breasts) presented for revision after implant placement for breast reconstruction, with the remaining 22 presenting for revision after implant placement for cosmetic indications. Average patient age was 47.9 years, and average BMI 24.3. Indications for surgery were implant malposition (37%), capsular contracture (30%), poor cosmesis/asymmetry (26%), and recurrent soft tissue ptosis (7%). Follow up averaged 355 days (range 174 to 799 days, SD=155). One patient experienced a seroma 2 weeks post-operatively, which resolved after a single aspiration. No infections, wound healing problems, or recurrences of implant malposition/capsular contracture were encountered.

**Conclusions:**

Durasorb PDO mesh appears to be efficacious as a breast implant pocket reinforcement in the reoperative setting with a low complication rate and durable results.

**Level of Evidence: 4:**

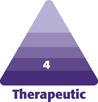

The number of cosmetic and reconstructive breast surgery procedures performed in the United States and worldwide has grown steadily over the preceding decade and so too has the number of opportunities for revisional breast surgery.^1^ Fundamentally, the vast majority of revisional breast surgery cases address some imbalance of the skin envelope and breast shape/size, which may be composed of native breast parenchyma or an implant. Controlling the position and shape of the breast mound is required to achieve a lasting and aesthetically pleasing result. However, experience in tissue expansion has conclusively demonstrated that forcing soft tissues to withstand constant mechanical stress (as would be produced by a heavy implant or a reshaped gland) will predictably lead to some degree of tissue expansion and produce undesirable changes in breast shape.

Proposed solutions to this problem have ranged from gland re-shaping in autologous breast surgery cases, to capsule manipulation,^2^ to the use of alloplastic devices such as meshes and dermal matrices^3,4^ to provide support. Bioabsorbable meshes used in the past have shown suboptimal results over the long term^5,6^ even when placed by experienced surgeons. Recent advances in materials engineering, however, have brought new options to market that promise to address the shortcomings of their predecessors.^7,8^ As these devices have found widespread use in breast reconstruction scenarios,^9^ bioabsorbable mesh is now also gaining acceptance in cosmetic breast surgery.^2,10-12^

Durasorb (Surgical Innovation Associates, Chicago, IL) is a new addition to the bioabsorbable mesh lineup. The device is a monofilamentous woven mesh composed of polydioxanone (PDO), which is also used in the more commonly encountered suture material. Durasorb offers the advantage of an intermediate bioabsorption time compared with polyglactin 910 or P4HB and thus aims to provide adequate duration of support to assure a lasting cosmetic result yet avoid the long-term complications of a slowly absorbing or permanent mesh. The current study described the authors’ experience with the use of this product in the setting of revisional breast implant surgery.

## METHODS

Approval for this investigation was obtained through an independent institutional review board (Western Institutional Review Board, Inc., Olympia, WA), and all guidelines of the Declaration of Helsinki were followed. The study was a prospective single-arm case series of adult females undergoing revisional breast surgery in the senior author’s practice (K.G.) who received Durasorb PDO mesh for implant or soft tissue support. Inclusion criteria were age > 18 years old, previous breast surgery (cosmetic or reconstructive), and need for revisional surgery. Patients were enrolled during the period between May 2019 and January 2021. The risks and benefits of using PDO mesh were explained to all patients deemed to be potential candidates for PDO mesh use based on the preoperative evaluation. Written consent was provided, by which the patients agreed to the use and analysis of their data. The decision to use Durasorb mesh intraoperatively was left to the surgeon’s discretion when it was deemed that additional soft tissue reinforcement was indicated beyond traditional capsule or soft tissue manipulation methods. Inset and fixation techniques were per the surgeon’s individual preference. All patients who received Durasorb were enrolled in the study. Patient demographics and clinical outcome data points were collected prospectively using the Medrio software package (San Francisco, CA) and analyzed using Excel (Microsoft, Redmond, WA). Clinical outcomes were tracked until the last patient follow-up and included any wound healing problems, seroma, hematoma, infection, recurrence of ptosis or implant malposition, or undesirable cosmetic result.

## RESULTS

A total of 17 patients were enrolled in the study, of whom 10 underwent bilateral procedures, for a total of 27 breasts receiving Durasorb mesh. Patient demographics are summarized in Table 1 and tracked clinical outcomes are summarized in Table 2. The average patient was 47.9 years old (range 28 to 73 years old), the average BMI was 24.3, and no patients were active smokers. Five patients had a history of mastectomy to the operated breast, with 1 of them having been previously radiated.

**Table 1.** Patient Demographics (n = 17 patients)

Patient characteristics	Average statistic or corresponding no. of patients in cohort
Age (years)	47.9 average (SD = 12.1)
BMI	24.3 average (SD = 4.2)
Smoking history Never smoker Former smoker (quit >1 year prior to surgery)	13 (76%) 4 (24%)
History of breast cancer?	5 (19%)
History of radiation to operated breast?	1 (4%)
Follow up (days)	355 average (SD = 155), range 174–799

BMI, body mass index; SD, standard deviation.

**Table 2.** Clinical Outcomes (n = 27 operated breasts)

Variable	Clinical outcome tracked	No. of patients	% of cohort
Indication for surgery	Implant malposition	10	37%
	Capsular contracture	8	30%
	Unacceptable cosmetic appearance	7	26%
	Soft tissue ptosis	2	7%
Procedure	Revision (complex)	15	56%
	Capsulorrhaphy	4	15%
	Implant pocket revision	6	22%
	Implant exchange and mastopexy	2	7%
Complications	Seroma	1	4%

The most common presenting complaint was implant malposition (10 of 27 breasts, 37%), followed by capsular contracture (8 of 27 breasts, 30%), unacceptable cosmetic appearance not otherwise specified (7 of 27 breasts, 26%), and recurrent soft tissue ptosis (2 of 27, 7%). The majority of patients underwent complex breast revisions (15 of 27 breasts, 56%)—patients were included in this group if multiple or complex surgical maneuvers were needed to accomplish the goals of surgery (eg, pocket revision as well as soft tissue revision, total vs partial capsulectomy). The remaining patients underwent implant pocket revision only (6 of 27 breasts, 22%), capsulorrhaphy (4 of 27 breasts, 15%), and 1 patient who underwent an implant exchange and primary mastopexy (2 of 17 breasts, 7%). The average follow-up time was 355 days, with the shortest follow-up in the series of 174 days and the longest of 799 days, with a median follow-up of 303 days.

**Figure 1.**
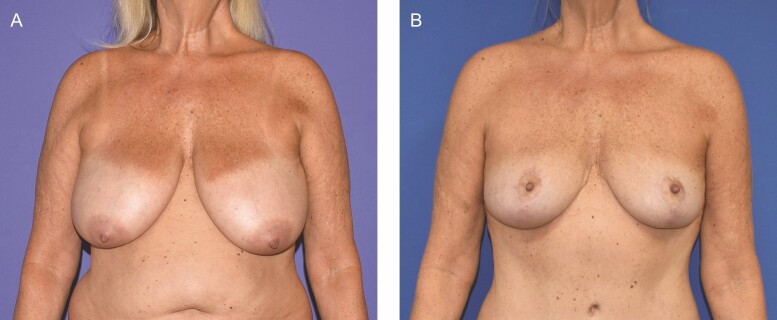
A 57-year-old otherwise healthy female undergoing inferior pedicle mastopexy, with bilateral soft tissue reinforcement using Durasorb mesh. (A) Preoperative appearance and (B) postoperative appearance at 8 months.

**Figure 2.**
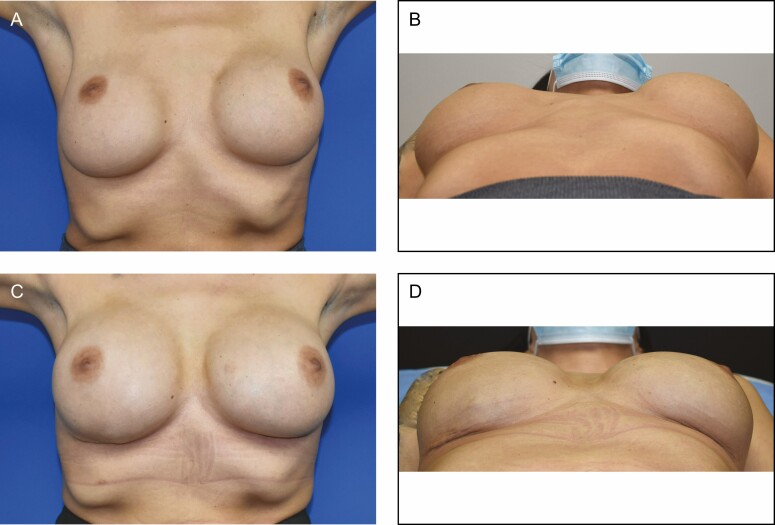
A 38-year-old female presenting with lateral displacement and horizontal malposition of her 15-year-old submuscular 350 cc saline implants. She underwent implant exchange to 450 cc silicone implants with pocket correction using Durasorb PDO mesh bilaterally. Results are shown at 6 months later with correction of displacement and malposition. (A) Preoperative appearance, anteroposterior (AP) view; (B) preoperative appearance, basal view; (C) postoperative appearance, AP view; (D) postoperative appearance, basal view.

Any implants placed were smooth, round, silicone-filled devices. The average implant size placed was 421 cc. In all cases, the Durasorb mesh was placed directly against the implant and secured using absorbable sutures. In all cases, the mesh was judged to be sufficient to definitively control implant position and breast footprint. Surgeon satisfaction with device pliability and ease of inset was subjectively judged as high. Figure 1 demonstrates the use of Durasorb mesh for breast parenchyma shaping and support in a 57-year-old otherwise healthy female who underwent inferior pedicle mastopexy with the removal of 425 g of tissue on the right and 610 g of tissue on the left. A durable result with adequate soft tissue support is seen at the 8-month postoperative mark. Figure 2 shows a case of a 38-year-old female who presented with lateral displacement and horizontal malposition of her 15-year-old submuscular 350 cc saline implants. She underwent implant exchange to 450 cc silicone implants with pocket correction using Durasorb PDO mesh bilaterally. Results are shown at 6 months later with correction of displacement and malposition.

No patients experienced wound healing complications or infection. One patient, a 73-year-old female undergoing revisional surgery for capsular contracture after breast augmentation, experienced a seroma at 2 weeks postoperatively. This was treated with a single aspiration in the clinic, with no recurrence. Aspirate cultures were negative, and the patient otherwise had an uneventful recovery. None of the patients experienced recurrence of ptosis, capsular contracture, or implant malposition at the time of the last follow-up.

## DISCUSSION

Capsular contracture remains the most common reason for revision after breast implant surgery, even in recent data,^13^ but size change, implant malposition, and cosmetic concerns are also among the top reasons for revision, with reoperation rates varying from 31.5% to as high as 46.0% for all indications combined.^14,15^ In almost all cases, the crux of the matter is some undesirable interaction between the implant and its soft tissue envelope. Overly exuberant soft tissue reaction/scarring may manifest as capsular contracture, whereas inadequate collagen deposition or soft tissue stretch may allow the force of gravity to displace the implant beyond its intended footprint.

Both synthetic mesh and acellular dermal matrix options promise surgeons an elegant method of controlling implant position in situations where the soft tissues are judged to have a high risk of failing to do so. The ideal product aims to be easy to use, have a low complication rate, and deliver on the promise of lasting implant support, all at a reasonable price.^16^ PDO mesh meets these requirements and presents an attractive option for plastic surgeons performing pocket revisions in reoperative breast surgery. The mesh is a monofilamentous construct and the constituent PDO offers intermediate in vivo durability of approximately 6 to 8 months in contrast to polyglactin 910 (2-4 months) and P4HB (18-24 months), combined with superior biocompatibility.^17^ This is in line with preclinical data demonstrating evidence of mesh incorporation at 1 month and resolution of ongoing foreign body reaction by the 12-month mark.^18-20^ The mid-range bioabsorption timeframe of PDO aims to strike the balance of providing mechanical support until the natural wound healing process is able to replace the mesh prosthesis with a stable capsule while avoiding long-term surgical site complications.^21^ The Durasorb (Surgical Innovation Associates, Chicago, IL) PDO mesh offers these advantages in a highly pliable, monofilament mesh designed to allow maximally fast tissue incorporation. The macroporous weave design poses no barrier to fluid egress, facilitating maximal contact between the mesh and overlying vascularized tissues. For these reasons, the authors do not routinely place drains with the types of cases illustrated in this series—the large pore size of the Durasorb mesh and its relatively small foreign body burden clinically do not seem to incite the same type of seromatous reaction as is common with an acellular dermal matrix (ADM), which presents a significantly greater amount of avascular substance the body needs to incorporate. At the current time, this rationale is conjecture based on surgical judgment, and we anticipate that future research in the biology of mesh/ADM incorporation/neovascularization will shed more light on the topic. 

Long-term clinical outcome studies have demonstrated the utility of acellular dermal matrices in prosthetic breast reconstruction and their continued widespread use attests to their clinical advantages.^22^ Synthetic mesh products have been by definition a more heterogenous group due to their different constituent polymers, yet they have overall seen increased adoption in the face of high ADM costs.^23-25^ The value proposition of the Durasorb PDO mesh is in the long-term stability of the breast implant pocket with a minimal complication profile and is demonstrated by the current pilot series. At the average follow-up of approximately 1 year, and up to 2 years in some of the earlier patients, no changes in implant position were noted by the authors or patients. Given that PDO mesh should no longer be effectively contributing to any breast implant support after approximately 8 to 12 months after implantation, we surmise that implant support thereafter is solely due to a stable soft tissue capsule. None of the patients in the current study have presented with complaints requiring reoperation and so no histologic or gross capsule analysis has yet been conducted, but the uniformly stable implant position in all patients at the last follow-up is a promising sign for the expected longevity of the results. The limitations of the implant pockets created by the soft tissue/Durasorb mesh interaction will become clear as patient outcomes continue to be tracked over longer durations.

Insofar as soft tissue support in breast reduction and mastopexy, the authors have found Durasorb mesh to be a valuable adjunct for 2 indications: shaping and constraining the pedicle/breast parenchyma in a more formal/thorough fashion when compared with using tacking or shaping sutures and providing support to the breast parenchyma in the interest of relieved downward pressure on the inferior skin flaps if these are less robust. These indications can be encountered in either primary or revision cases, and at this point, the mesh becomes like any other instrument in the surgeon’s hands—to be used when judgment dictates it. 

The series presented herein represents the first published data on the use of Durasorb PDO mesh in breast implant revision/cosmetic breast surgery, complementing the existing series on the use of this product in primary breast reconstruction. This study has obvious limitations due to its design as a single surgeon series and a small sample size. As much as the heterogeneous nature of the cohort demonstrates the versatility of the Durasorb mesh, it also precludes any statistical analysis given the small cohort. As this is a relatively new product, follow-up is also limited, so the long-term (>2 years) complication and recurrence data are not yet available. In the same vein, while there are clinical data to suggest the efficacy of synthetic mesh in breast surgery,^7,8,11^ there are no histological data from in vivo use cases for Durasorb yet. Similarly, the additional tensile strength due to the mesh cannot be quantified in the in vivo setting. As time goes on and more data are gathered, we would hope that opportunities will arise for more in-depth histologic studies of the resulting capsules to objectively define the contribution of the bioresorbable mesh.

## CONCLUSIONS

PDO mesh demonstrates high biocompatibility, with no major adverse events observed in this series. At the time of last follow-up of up to 2 years, no patient demonstrated recurrence of implant malposition, capsular contracture, or recurrence of ptosis. Durasorb PDO mesh shows promise as a method of soft tissue reinforcement and demonstrates clinically stable implant capsules past the point of presumed mesh resorption. Further investigation with larger cohorts will be needed to delineate the limitations and long-term outcomes in the use of this mesh in reoperative breast implant surgery.

## References

[1] The Aesthetic Society’s Cosmetic Surgery National Data Bank: Statistics 2020. Aesthet Surg J. 2021;41(2):1-16. 10.1093/asj/sjaa144 32542351

[2] Calobrace MB, Mays C, Wilson R, Wermeling R. Popcorn capsulorrhaphy in revision aesthetic breast surgery. Aesthet Surg J. 2020;40(1):63-74. doi:10.1093/asj/sjy3244 30544132

[3] Slavin SA, Lin SJ. The use of acellular dermal matrices in revisional breast reconstruction. Plast Reconstr Surg. 2012;130(5 Suppl 2):70s-85s. doi:10.1097/PRS.0b013e31825f23ca 23096989

[4] Maxwell GP, Gabriel A. Efficacy of acellular dermal matrices in revisionary aesthetic breast surgery: a 6-year experience. Aesthet Surg J. 2013;33(3):389-399. doi:10.1177/1090820X134789677 23439064

[5] Góes JC . Periareolar mammaplasty: double skin technique with application of polyglactine or mixed mesh. Plast Reconstr Surg. 1996;97(5):959-968. doi:10.1097/00006534-199604001-000122 8618999

[6] Goes JC, Bates D. Periareolar mastopexy with FortaPerm. Aesthetic Plast Surg. 2010;34(3):350-358. doi:10.1007/s00266-009-9462-55 20336456

[7] Adams WP, Jr, Baxter R, Glicksman C, Mast BA, Tantillo M, Van Natta BW. The use of poly-4-hydroxybutyrate (P4HB) scaffold in the ptotic breast: a multicenter clinical study. Aesthet Surg J. 2018;38(5):502-518. doi:10.1093/asj/sjy022 29401215

[8] Adams WP, Jr, Toriumi DM, Van Natta BW. Clinical use of GalaFLEX in facial and breast cosmetic plastic surgery. Aesthet Surg J. 2016;36(suppl_2):S23-S32. doi:10.1093/asj/sjw142 27697886

[9] Faulkner HR, Shikowitz-Behr L, McLeod M, Wright E, Hulsen J, Austen WG, Jr. The use of absorbable mesh in implant-based breast reconstruction: a 7-year review. Plast Reconstr Surg. 2020;146(6):731e-736e. doi:10.1097/PRS.00000000000073844 PMC7676463 33234950

[10] Qureshi AA, Myckatyn TM, Tenenbaum MM. Mastopexy and mastopexy-augmentation. Aesthet Surg J. 2018;38(4): 374-384. doi:10.1093/asj/sjx1811 29365038

[11] Becker H . Update on the use of synthetic mesh in reconstructive and cosmetic breast surgery. Aesthetic Plast Surg. 2020;44(4):1128-1129. doi:10.1007/s00266-020-01767-22 32766897

[12] József Z, Újhelyi M, Ping O, et al. Long-term dynamic changes in cosmetic outcomes and patient satisfaction after implant-based postmastectomy breast reconstruction and contralateral mastopexy with or without an ultrapro mesh sling used for the inner bra technique. A retrospective correlational study. Cancers (Basel). 2020;13(1):73. doi:10.3390/cancers13010073 33383874 PMC7795018

[13] Coroneos CJ, Selber JC, Offodile AC, Butler CE, Clemens MW. US FDA breast implant postapproval studies: long-term outcomes in 99,993 patients. Ann Surg. 2019;269(1):30-36. doi:10.1097/SLA.00000000000029900 30222598

[14] Stevens WG, Calobrace MB, Alizadeh K, Zeidler KR, Harrington JL, d′Incelli RC. Ten-year core study data for Sientra’s Food and Drug Administration-approved round and shaped breast implants with cohesive silicone gel. Plast Reconstr Surg. 2018;141(4S Sientra Shaped and Round Cohesive Gel Implants):7s-19s. doi:10.1097/prs.0000000000004350 29595714

[15] Spear SL, Murphy DK. Natrelle round silicone breast implants: core study results at 10 years. Plast Reconstr Surg. 2014;133(6):1354-1361. doi:10.1097/PRS.00000000000000211 24867717 PMC4819531

[16] Colwell AS, Taylor EM. Recent advances in implant-based breast reconstruction. Plast Reconstr Surg. 2020;145(2):421e-432e. doi:10.1097/PRS.00000000000065100 31985660

[17] Fatkhudinov T, Tsedik L, Arutyunyan I, et al. Evaluation of resorbable polydioxanone and polyglycolic acid meshes in a rat model of ventral hernia repair. J Biomed Mater Res B Appl Biomater. 2019;107(3):652-663. doi:10.1002/jbm.b.341588 30091512 PMC6585936

[18] Mlodinow AS, Yerneni K, Hasse ME, Cruikshank T, Kuzycz MJ, Ellis MF. Evaluation of a novel absorbable mesh in a porcine model of abdominal wall repair. Plast Reconstr Surg Glob Open. 2021;9(5):e3529. doi:10.1097/gox.0000000000003529 34881139 PMC8647887

[19] Tang H, Xu Z, Qin X, et al. Chest wall reconstruction in a canine model using polydioxanone mesh, demineralized bone matrix and bone marrow stromal cells. Biomaterials. 2009;30(19):3224-3233. doi:10.1016/j.biomaterials.2009.02.007 19233465

[20] Goonoo N, Jeetah R, Bhaw-Luximon A, Jhurry D. Polydioxanone-based bio-materials for tissue engineering and drug/gene delivery applications. Eur J Pharm Biopharm. 2015;97:371-391. doi:10.1016/j.ejpb.2015.05.024 26614558

[21] Williams SF, Martin DP, Moses AC. The history of GalaFLEX P4HB scaffold. Aesthet Surg J. 2016;36(suppl 2):S33-S42. doi:10.1093/asj/sjw1411 27697885 PMC5070449

[22] Scheflan M, Colwell AS. Tissue reinforcement in implant-based breast reconstruction. Plast Reconstruct Surg Glob Open. 2014;2(8):e192. doi:10.1097/GOX.00000000000001400 PMC4236353 25426375

[23] Kim JYS, Mlodinow AS. What’s new in acellular dermal matrix and soft-tissue support for prosthetic breast reconstruction. Plast Reconstr Surg. 2017;140(5S Advances in Breast Reconstruction):30s-43s. doi:10.1097/prs.0000000000003950 29064920

[24] Dieterich M, Paepke S, Zwiefel K, et al. Implant-based breast reconstruction using a titanium-coated polypropylene mesh (TiLOOP Bra): a multicenter study of 231 cases. Plast Reconstr Surg. 2013;132(1):8e-19e. doi:10.1097/PRS.0b013e318290f8a00 23806958

[25] Tessler O, Reish RG, Maman DY, Smith BL, Austen WG, Jr. Beyond biologics: absorbable mesh as a low-cost, low-complication sling for implant-based breast reconstruction. Plast Reconstr Surg. 2014;133(2):90e-99e. doi:10.1097/01.prs.0000437253.55457.633 24469217

